# Differentiation of Thyroid Nodules Difficult to Diagnose With Contrast-Enhanced Ultrasonography and Real-Time Elastography

**DOI:** 10.3389/fonc.2020.00112

**Published:** 2020-02-27

**Authors:** Xuehua Xi, Luying Gao, Qiong Wu, Shibao Fang, Jingzhu Xu, Ruyu Liu, Xiao Yang, Shenling Zhu, Ruina Zhao, Xingjian Lai, Xiaoyan Zhang, Bo Zhang, Yuxin Jiang

**Affiliations:** ^1^Department of Ultrasound, China–Japan Friendship Hospital, Beijing, China; ^2^Department of Ultrasound, Peking Union Medical College Hospital, Chinese Academy of Medical Sciences & Peking Union Medical College, Beijing, China; ^3^Department of Ultrasound, Affiliated Hospital of Qingdao University, Qingdao, China; ^4^Department of General Surgery, Peking Union Medical College Hospital, Chinese Academy of Medical Sciences & Peking Union Medical College, Beijing, China

**Keywords:** thyroid carcinoma, contrast-enhanced ultrasonography, elastosonography, American Thyroid Association, intermediate- and low-suspicion patterns

## Abstract

According to the 2015 American Thyroid Association (ATA), referred risk stratification and thyroid nodules with intermediate- and low-suspicion patterns are difficult to diagnose. The objective of this study is to evaluate the diagnostic performance of contrast-enhanced ultrasonography (CEUS) and elastosonography (ES) for the differentiation of these thyroid nodules. From November 2011 to June 2016, a total of 163 thyroid nodules with intermediate- and low-suspicion patterns in 150 consecutive patients at our hospital were studied before surgery. With surgical pathology as the standard, the diagnostic value of CEUS and ES was analyzed. There were 29 (17.8%) malignant lesions and 134 (82.2%) benign lesions. The enhancement patterns of CEUS, the echogenicity, and the elastography were significantly different between malignant and benign lesions (*P* < 0.05). Heterogenous enhancement was more common in malignant nodules, and the sensitivity, specificity, positive predictive value, negative predictive value, and odds ratio were 51.7, 88.1, 48.4, 89.4, and 10.1%, respectively. The diagnostic accuracy of CEUS was better than the conventional ultrasound [area under the curve (AUC), 0.729 vs. 0.616, *P* = 0.021]. The enhancement patterns of CEUS were helpful in the differential diagnosis of thyroid nodules with intermediate and low suspicion.

## Introduction

The 2015 American Thyroid Association Management Guidelines for Adult Patients with Thyroid Nodules and Differentiated Thyroid Cancer (1) (2015 ATA guidelines) proposed a risk stratification of thyroid nodules based on a series of studies on the ultrasonographic features of thyroid nodules. The guide classifies the sonographic appearance of the vast majority of thyroid nodules into the following categories of ultrasound patterns: high suspicion, intermediate suspicion, low suspicion, very low suspicion, and benign. Intermediate suspicion (malignancy risk 10–20%) nodules are hypoechoic solid nodules with smooth regular margins and without malignant features such as microcalcifications, extrathyroidal extension, or a taller-than-wide shape. Low-suspicion (malignancy risk, 5–10%) nodules are isoechoic or hyperechoic solid nodules or partially cystic nodules with eccentric uniformly solid areas without malignant features. These nodules have similar ultrasound features as nodules suspicious of follicular lesions in the “British Thyroid Association Guidelines for the Management of Thyroid Cancer” (2) (BTA guidelines). Despite the fact that the malignancy risk is not relatively high, differentiating malignant from benign lesions by conventional ultrasonography (CUS) is challenging.

Contrast-enhanced ultrasonography (CEUS) has been widely used to evaluate the microvessel perfusion of thyroid nodules. Elastosonography (ES) quantifies the firmness of the tissue and displays it as a color map on which the hardness of the tissues can be reflected. Many studies have concluded that the accuracy of the diagnosis of thyroid nodules can be increased by combining CEUS and ES with CUS (3–5). However, the value of CEUS and ES in evaluating nodules with intermediate- and low-suspicion patterns is unclear.

## Methods

This study was approved by the ethics committee of Peking Union Medical College Hospital (PUMCH), and informed consent was obtained from all patients before CEUS and ES.

### Patients

A total of 604 patients with thyroid nodules admitted to PUMCH from November 2011 to June 2016 who met the following criteria were included in this study: (a) CUS indicated that the thyroid nodule was of intermediate or low suspicion according to the 2015 ATA guidelines for referred risk stratification, and (b) the largest diameter of the nodule was larger than 5 mm in size. The exclusion criteria were as follows: (a) patients who did not undergo surgery, (b) patients younger than 18 years of age, (c) women during pregnancy or lactation, and (d) patients who could not tolerate the intravenous injection of contrast agents because of heart, lung, kidney, or other vital organ dysfunction or severe allergies. A total of 163 thyroid nodules in 150 consecutive patients (mean age, 47.9; range, 18–83 years) were included. Two nodules in eight patients each and three nodules in three patients each were included. In six of those eight patients, the two nodules were located in different lobes. In two other patients, the two nodules were not near each other, although they were in the same lobe. In three patients, two of the three nodules were located in the same lobe, and the other was located in a different lobe. CUS, CEUS, and ES were performed in all patients, and the ultrasound-guided fine needle aspiration (FNA) was performed in nine patients before surgery.

### Ultrasound Examination

All ultrasound (US) examinations were performed with a 5- to 12-MHz liner probe (iU22; Philips Medical System, Bothell, WA, United States). The CUS examination was performed with the standard equipment settings for thyroid glands. Standard machine settings for CEUS were used, and the contrast medium used was SonoVue (Bracco Imaging, Milan, Italy). With a 20- or 22-gauge peripheral intravenous cannula, SonoVue was injected intravenously as a bolus at a dose of 1.2 ml, followed by 5 ml of normal saline as a flush. The timer on the US machine was then started, and each contrast imaging acquisition lasted more than 2 min after the bolus injection; the imaging was digitally stored. During real-time elastography (RTE), the probe was positioned perpendicular to the skin, and no compression was applied at the skin above the targeted thyroid nodule. When there were two nodules in a patient, CEUS was performed on the second nodule after the first injected contrast agent was completely cleaned in the whole gland. CUS, RTE, and CEUS images and cine clips were analyzed by two radiologists with more than 5 years of experience in thyroid US. They were blinded to the patients' clinical data and pathological results. In cases of discrepancies between the two readers, a consensus was reached after discussion.

The CUS features of all thyroid nodules were recorded according to the halo, echogenicity, internal component, echotexture (homogenous or heterogenous), calcifications, and vascularity. Halos were classified as absent, regularly thin, or irregular (including thick halos, incomplete halos, and halos of different widths). Echogenicity was classified as hypoechogenicity, isoechogenicity, hyperechogenicity, or marked hyperechogenicity relative to the surrounding normal parenchyma. The internal component of a nodule was classified as solid (defined as composed entirely or nearly entirely of soft tissue, with only a few tiny cystic spaces) (6), predominantly solid, and predominantly cystic. Calcifications, when present, were categorized as microcalcifications, disrupted rim calcifications, and other types of calcifications. Microcalcifications were defined as calcifications that were ≤1 mm in diameter and visualized as tiny hyperechoic foci. Disrupted rim calcifications were defined as interrupted peripheral calcifications in association with a soft tissue rim outside the calcification. When a nodule had both microcalcifications and other types of calcifications, it was classified as having microcalcifications. The vascularity of nodules was classified into five types by Frates (7) as follows: 0 for no visible flow, 1 for minimal internal flow without a peripheral ring, 2 for a peripheral ring of flow (defined as >25% of the nodule's circumference) with minimal or no internal flow, 3 for a peripheral ring of flow with a small to moderate amount of internal flow, and 4 for extensive internal flow with or without a peripheral ring. We regarded type 4 as increased intranodular vascularity. CEUS characteristics included peak intensity (categorized as low, equal, or high) and enhanced pattern (categorized as homogenous, heterogenous, or ring enhancing). Elastography score elasticity was classified in five different patterns, adding elastography score (ESS) 0 to the version of Asteria criteria (8): ESS 0, red and blue, or blue and green, or red and green are layered distribution in cystic nodules or predominantly cystic nodules; ESS 1, homogenously in green (soft); ESS 2, predominantly in green with few blue areas/spots; ESS 3, predominantly in blue with a few green areas/spots; and ESS 4, completely in blue (hard).

### Review of Histopathology

An experienced pathologist who was blinded to the clinical information reviewed the histopathology of all the specimens. The papillary thyroid carcinomas (PTCs) were divided into classical PTC and follicular variants of the PTC (FVPTC).

### Statistical Analysis

The statistical analysis was performed with the SPSS statistical package (Version 19.0, SPSS Chicago, IL, United States) and MedCalc 11.4.2.0 software (MedCalc Software, Ostend, Belgium). The quantitative data were expressed as the mean ± standard deviation. The Student's *t*-test was used for comparing the groups. The χ^2^ test or Fisher's exact test was used to compare categorical data. The sensitivity, specificity, positive predictive value (PPV), negative predictive value (NPV), and accuracy were calculated through a comparison with the pathological findings. A receiver operating characteristic (ROC) curve analysis was used to compare CEUS, ES, and CUS. A *P* < 0.05 was considered statistically significant.

## Results

### Clinical Characteristics

The clinical characteristics are summarized in Table 1. The age, gender, nodule size, and incidence of coexistence with Hashimoto thyroiditis (HT) did not significantly differ between the benign and malignant groups (*P* > 0.05). The results of the cytopathology and histopathology of the nine nodules that underwent FNA are summarized in Table 2. Histological pathology demonstrated that 29 lesions (17.8%) were malignant, and 134 lesions (82.2%) were benign. One hundred one nodular goiters (75.4%) constituted the majority of benign lesions, 18 of the 134 lesions were adenomatous nodules (ANs). The remainder of the benign lesions consisted of six follicular adenomas (FAs, 25.4%) and nine HTs (6.7%). Of the malignant lesions, 21 cases were PTCs, with 17 classical variant and 4 follicular variants, 7 cases were follicular carcinomas (FCs), and 1 case was medullary carcinoma (MC). Cervical lymph node metastasis was confirmed in one FC and six PTCs. Metastatic lesions were found in cervical striated muscles and fibrous connective tissues in another FC.

**Table 1:** Clinical characteristics of nodules with intermediate and low suspicion.

**Clinical characteristics**	**Malignant group (*n* = 29)**	**Benign group (*n* = 121)**	***P*-value**
Age (years)	44.0 ± 11.9	48.7 ± 11.8	0.706
Sex			0.054
Female	17 (58.6)	91 (75.2)	
Male	12 (41.4)	30 (24.8)	
Mixed with HT	1 (3.4)	19 (15.7)	0.081
Size (cm)^*^	2.1 ± 1.5	2.6 ± 1.4	0.675

*HT, Hashimoto thyroiditis*.

* *For 163 nodules*.

**Table 2:** Comparison of fine needle aspiration (FNA) cytopathology and histological pathology of nine nodules.

**Nodule number**	**FNA cytopathology**	**Histological pathology**
1	A small amount of thyroid follicular epithelial cells and no tumor cells	Thyroid atypical adenoma
2	Thyroid follicular epithelial cells and no tumor cells	Thyroid adenoma
3	Suspicious for a follicular neoplasm	Thyroid follicular carcinoma
4	Thyroid follicular epithelial cells and no tumor cells	Thyroid follicular carcinoma
5	No exception of follicular adenoma	Nodular goiter with adenomatous hyperplasia
6	Thyroid follicular adenoma	Nodular goiter with adenomatous hyperplasia
7	Tumor-like lesion from thyroid follicular epithelial cells. Tumor or adenomatous change cannot be distinguished because no specific capsule was seen.	Nodular hashimoto thyroiditis
8	Consistent with thyroid follicular neoplasm, and thyroid adenoma was considered	Nodular goiter with adenomatous hyperplasia
9	Thyroid follicular epithelial cells and no tumor cells	Nodular goiter

### US Characteristics

The CUS characteristics are summarized in Table 3. No significant difference was observed in the US features of absent or irregular halos (a solid internal component, a heterogenous echo texture, and microcalcification or disrupted rim calcification between benign and malignant lesions). Moderate or abundant blood flow (Frates type 3 or 4) was detected in the majority of the nodules with intermediate- and low-suspicion patterns. The difference in increased intranodular vascularity (Frates type 4) between benign and malignant lesions was not statistically significant (*P* > 0.05). Of the malignant nodules, two PTCs showed peripheral vascularity without intranodular vascularity, and four PTCs showed partial hypervascularity with perforating vessels into the nodule. Regular peripheral and intranodular vascularity was found in the other 15 malignant lesions.

**Table 3:** Ultrasound (US) characteristics of benign and malignant thyroid nodules with intermediate and low suspicion.

**US characteristics**	**Malignant nodules (*n* = 29)**	**Benign nodules (*n* = 134)**	***P-*value**
Halo			0.183
Absent	17 (58.6)	58 (43.3)	
Regular and thin	8 (27.6)	62 (46.3)	
Irregular	4 (13.8)	14 (10.4)	
Internal component			0.157
Solid	20 (69.0)	74 (55.2)	
Predominantly solid	9 (31.0)	47 (35.1)	
Predominantly cystic	0 (–)	13 (9.7)	
Echogenicity			0.033
Hyperechogenicity	1 (3.4)	18(13.4)	
Isoechogenicity	5 (17.2)	53 (39.6)	
Hypoechogenicity	20 (69.0)	57 (42.5)	
Marked hypoechogenicity	3 (10.3)	6 (4.5)	
Homogeneity			0.762
Homogenous	7 (24.1)	36 (26.9)	
Heterogenous	22 (75.9)	98 (73.1)	
Calcification			0.746
Absent	18 (62.1)	95 (70.9)	
Microcalcification	1 (3.4)	3 (2.2)	
Disrupted rim calcification	0 (0)	0 (0)	
Other types of calcification	10 (34.5)	36 (26.9)	
Vascularity type			0.429
Type 0	0 (0)	0 (0)	
Type 1	2 (6.9)	0 (-)	
Type 2	3 (10.3)	1 (0.7)	
Type 3	4 (13.8)	27 (20.1)	
Type 4	20 (69.0)	106 (79.1)	
Risk stratification			<0.001
Intermediate suspicion	21 (72.4)	45 (33.6)	
Low suspicion	8 (27.6)	89 (66.4)	

**Figure 1:**
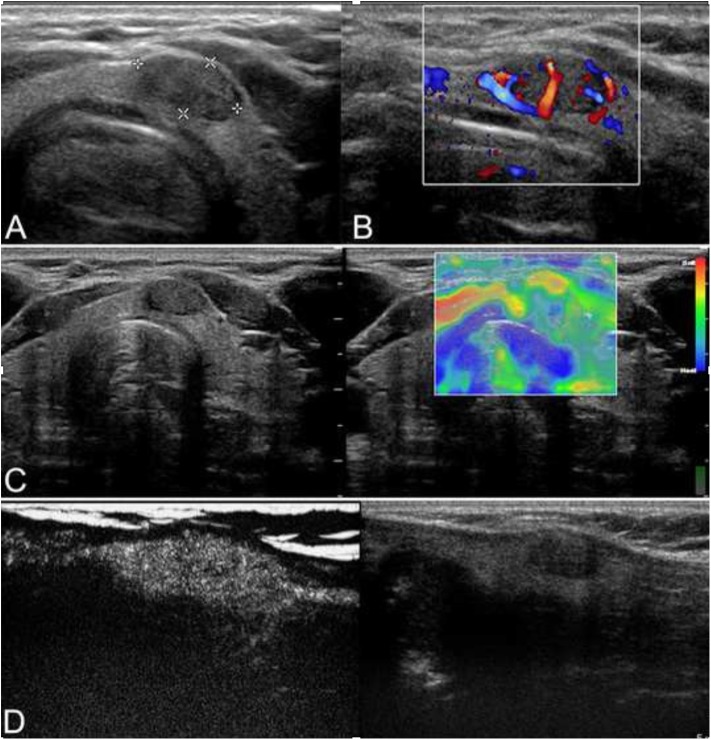
Ultrasound of a 42-year-old man who incidentally detected a thyroid nodule is shown. **(A)** Conventional ultrasonography (CUS) showed that there was a solid hypoechoic nodule with a regular margin in the isthmus of the thyroid. The nodule was of intermediate suspicion. **(B)** Color Doppler showed intranodular and peripheral vascularity. **(C)** The elastography score was 2, indicating that the nodule was soft. **(D)** Contrast-enhanced ultrasonography (CEUS) revealed heterogenous enhancement. The nodule was a papillary thyroid carcinoma (PTC) confirmed by histological pathology.

**Figure 2:**
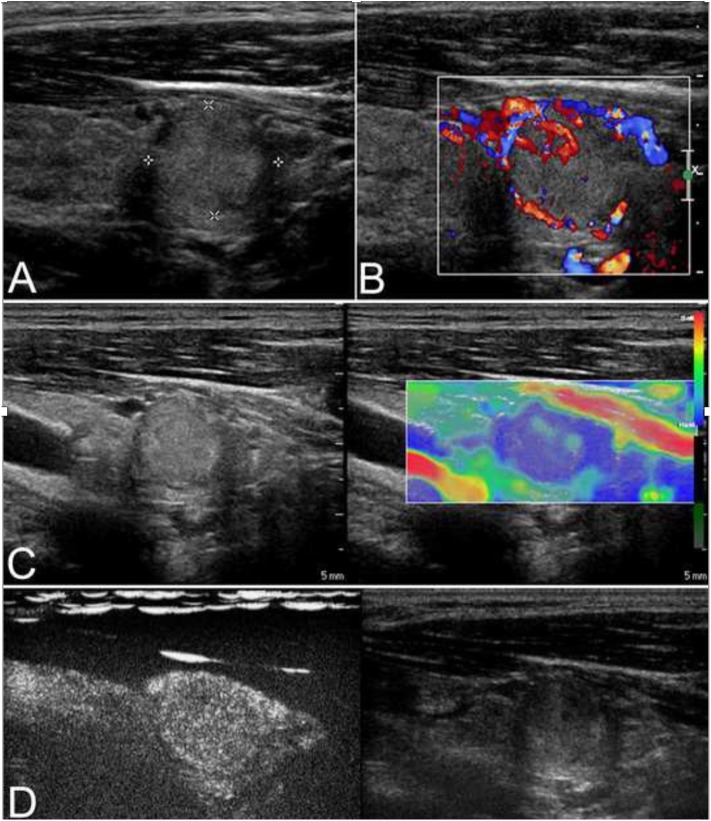
This case is a 27-year-old man with an follicular carcinoma (FC) nodule. **(A)** Conventional ultrasonography (CUS) showed a solid hyperechoic nodule in the left lobe that was associated with a regular thin halo and an inside area that was hypoechoic, which is indicative of low suspicion. **(B)** Abundant intranodular and peripheral vascularity was detected. **(C)** The elastography score was 2, indicating a soft stiffness. **(D)** Contrast-enhanced ultrasonography (CEUS) revealed heterogenous enhancement.

**Figure 3:**
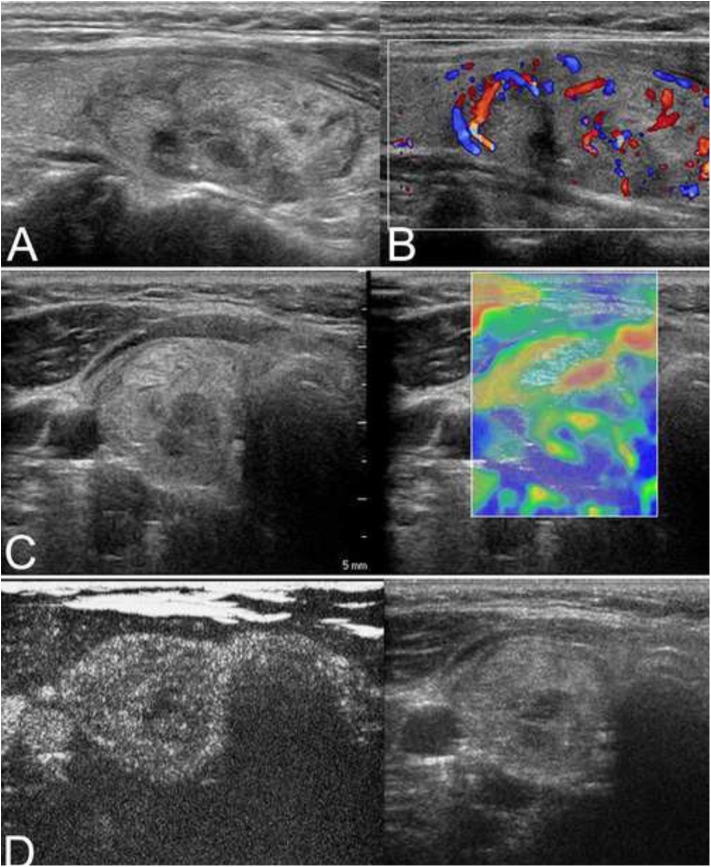
**(A)** The isoechoic solid nodule with a regular thin halo was evaluated as low suspicion by conventional ultrasonography (CUS) in a 38-year-old man. **(B)** Color Doppler showed intranodular and peripheral vascularity. **(C)** The elastography score was 3, indicating a hard stiffness. **(D)** Contrast-enhanced ultrasonography (CEUS) revealed ring enhancement. The nodule was a follicular adenoma.

**Figure 4:**
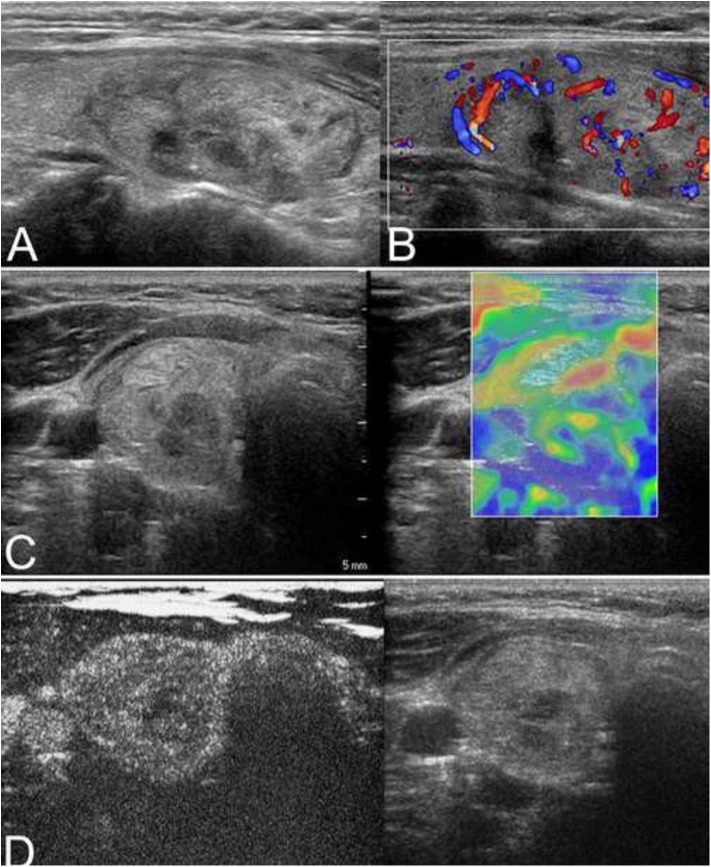
A nodular goiter with adenomatous hyperplasia in a 59-year-old woman is shown. **(A)** Conventional ultrasonography (CUS) showed that the solid hyperechoic nodule in the right lobe was heterogenous in echotexture and had regular margins, indicative of low suspicion. **(B)** Color Doppler showed intranodular and peripheral vascularity. **(C)** The elastography score was 2, indicating a soft stiffness. **(D)** Contrast-enhanced ultrasonography (CEUS) revealed ring-enhancement.

The CEUS characteristics and ES of the nodules are shown in Table 4 and Figures 1–4. Heterogenous enhancement was significantly (*P* < 0.001) associated with a malignant outcome with a 10.1 odds ratio [OR; 95% confidence interval (CI) ranging from 3.9 to 25.7]. The sensitivity, specificity, positive predictive value, and negative predictive value were 51.7, 88.1, 48.4, and 89.4%, respectively. The enhancement patterns of 12 PTCs and 2 FCs and 1 MC were heterogenous, 2 PTCs were homogenous, 1 PTC had no enhancement, and the remaining 6 PTCs and 5 FCs exhibited a ring-enhancing pattern. Among the benign nodules, 89 cases (66.4%) showed a ring-enhancing pattern; 24 cases (17.9%) showed homogenous enhancement, including 18 nodular goiters and 3 HT; and 16 cases (11.9%) showed heterogenous enhancement, including 10 nodular goiter, 2 FAs, and 4 HTs. The intensity of enhancement was not significantly different (*P* = 0.289). Most of the nodules showed soft texture in elasticity imaging, and the difference in the ES between the benign and malignant groups was significant (*P* = 0.015).

**Table 4:** Contrast-enhanced ultrasonography (CEUS) and elastosonography (ES) characteristics of malignant and benign thyroid nodules.

**CEUS and ES characteristics**	**Malignant nodules (*n* = 29)**	**Benign nodules (*n* = 134)**	***P-*value**
Enhanced pattern			<0.001
Ring enhancing	11 (37.9)	89 (66.4)	
Homogenous	2 (6.9)	24 (17.9)	
Heterogenous	15 (51.7)	16 (11.9)	
No enhancement	1 (3.4)	5 (3.7)	
Peak intensity^*^			0.289
High	10 (34.5)	55 (41.0)	
Equal	9 (31.0)	51 (38.1)	
Low	9 (31.0)	21 (15.7)	
Elastography score^†^			0.015
0	1 (3.8)	13 (9.7)	
1	0 (–)	12 (9.0)	
2	11 (42.3)	68 (50.7)	
3	9 (34.6)	36 (26.9)	
4	5 (19.2)	5 (3.7)	

* *The peak intensity of two nodules was not determined because the nodules were too large and the surrounding thyroid parenchyma could not be displayed in the same plane for reference*.

† *The ES of the three nodules was not determined because the nodules were too close to the carotid artery and the results were unreliable*.

### Diagnostic Performance of the CEUS and ES

Sensitivity, specificity, PPV, NPV, accuracy, and area under the curve (AUC) of CUS were 69.0, 57.5, 26.0, 89.5, 59.5, and 0.61% (95% CI, 0.53–0.69), respectively. Nodules with the CEUS patterns of heterogenous enhancement was considered malignant. The sensitivity, specificity, PPV, NPV, accuracy, and AUC for CEUS were 51.7, 88.1, 48.4, 89.4, 81.6, and 0.73% (95% CI, 0.65–0.80), respectively. The ROC curves demonstrated that the best cutoff value for ES was 3. The sensitivity, specificity, PPV, NPV, accuracy, and AUC for ES were 53.8, 69.4, 25.5, 88.6, 66.9, and 0.62% (95% CI, 0.54–0.69), respectively (Table 5). The CEUS showed a higher AUC than CEUS and ES (0.729 vs. 0.616, *P* = 0.021; 0.729 vs. 0.608, *P* = 0.046). We added this in *Result* and *Abstract* and emphasized in red color (lines 37 and 88–99).

**Table 5:** Diagnostic efficiency of the contrast-enhanced ultrasonography (CEUS), elastosonography (SE) and conventional ultrasonography (CUS).

	**Se (%)**	**Sp (%)**	**PPV (%)**	**NPV (%)**	**Ac (%)**	AUC (95% CI)*
CUES	51.7	88.1	48.4	89.4	81.6	0.729 (0.653–0.796)
SE	53.8	69.4	25.5	88.6	66.9	0.616 (0.536–0.692)
CUS	69.0	57.5	26.0	89.5	59.5	0.608 (0.528–0.685)

*CEUS, contrast-enhanced ultrasonography; ES, elastosonography; CUS, conventional ultrasonography; Se, sensitivity; Sp, specificity; PPV, positive predictive value; NPV, negative predictive value; AUC, area under the curve; CI, confidence interval*.

## Discussion

High-resolution neck ultrasonography is the most important method for the evaluation of a thyroid nodule. The latest ATA guidelines have proposed criteria for risk stratification that categorizes the thyroid nodules and stratify the risk of malignancy. However, the differential diagnosis of nodules with intermediate and low suspicion is difficult. In the “British Thyroid Association Guidelines for the Management of Thyroid Cancer” (2), these nodules are defined as suspicious of follicular lesions on ultrasound and classified as indeterminate/equivocal. A total of 71.4% of the nodules were thyroid tumors with a follicular growth pattern. Our study showed that the enhancement pattern was a significant factor for differentiating benign and malignant nodules with intermediate and low suspicion. Some researchers have revealed that age, sex, and tumor size are predictive parameters of malignancy in follicular neoplasms of the thyroid (diagnosed by intraoperative frozen section or FNA) (9, 10). The male gender, ages <45 years, and nodules larger than 4 cm were risk factors for malignancy. In our study, 15.7% of women and 28.6% of men had malignant nodules. Sixteen patients (10.7%) younger than 45 years of age and 13 patients (8.7%) older than 45 years of age were diagnosed with malignant lesions, with no statistically significant differences observed. Of the 29 malignant nodules, only 3 (10.3%) were larger than 4 cm. Therefore, using sex, age, and nodule size as the predictive malignant parameters may result in missed diagnoses and misdiagnoses.

Conventional ultrasound features such as a solid internal component, hypoechogenicity, irregular margins, a taller-than-wide shape, and microcalcification are predictive of thyroid carcinoma (11, 12). However, these features are classical malignant characteristics for non-follicular neoplasms. Koike et al. (13) found that the sensitivity of preoperative US diagnosis was 18.2% for follicular neoplasms. Multiple logistic regression analyses revealed that conventional ultrasound could not identify FC and FA (13). The results of this study were similar to these previous results.

CEUS can reflect the state of microcirculation perfusion of nodules. Three meta-analyses showed that both the sensitivity and specificity of CEUS were more than 85% (14–16). Most research have suggested that a heterogenous enhancement pattern is a reliable index for predicting malignancy and that a ring-enhancement pattern is a feature of benign nodules. Our study has revealed that the predictivity of malignancy of ATA risk stratification is improved by adding CEUS. The malignancy risk of the intermediate and low suspicious nodules is 5–20%. The risk can increase to 38.5% when the nodule shows heterogenous enhancement. Despite the low sensitivity (51.7%), it has a very high specificity and OR for malignancy. Of the 29 malignant nodules, 12 PTCs and 2 FCs and MC presented with heterogenous enhancement, which might be due to the more heterogenous growth of malignant nodules and the more imbalanced blood distribution, with the coexistence of areas of rich and poor microvasculature. In addition, malignant nodules contain regions of complicated morphological collagen degeneration, which have either no small vessels or vessels whose caliber is so small that microbubbles cannot enter, resulting in heterogenous enhancement. However, the other six PTCs and five FCs presented with ring enhancement. Two FVPTC nodules were 3.8 and 4.0 cm in ultrasound images, but the pathological size of the cancer area was only 0.4 and 0.5 cm, respectively, with the surrounding tissue presenting with nodular goiter. This finding may result in a benign enhancement pattern. The reason that malignant nodules showed ring enhancement may be that the nodules were surrounded by vessels or that vessels existed in the capsule of the nodule, which is consistent with the color Doppler features.

Elastosonography provided an estimation of tissue stiffness. In our study, only 14 malignant nodules were hard, with 3–4 elastography scores. The other 15 malignant nodules were relatively soft. This result was different from most studies, which reported that malignant thyroid nodules were often stiff. The most likely reason is that the constitution of the cases was vastly different. In this study, 24.1% of the malignant nodules were FCs, and 13.8% of the nodules were FVPTCs. Pathological changes in these nodules showed that follicular cells containing colloid were abundant, making the texture soft. Therefore, real-time elastography was not able to distinguish benign and malignant nodules with intermediate and low suspicion.

FNA is the best preoperative diagnostic method for thyroid nodules but is indeterminate in 15–20% of the cases especially when follicular neoplasms are involved. When a follicular neoplasm is suspected, the histological possibilities include an AN, FA, or FC. FVPTC will also sometimes fall into this category when the nuclear features are more subtle. These features cannot be distinguished by the use of cytology alone because the evaluation of morphology is subjective (17, 18) and the sample is too limited to represent the entire heterogenous lesion. In addition, the presence of capsule or vessel invasion for diagnosing FC cannot be evaluated by FNA (19). One FC in this study was suspicious for a follicular neoplasm, and the other FC exhibited only thyroid follicular epithelial cells and no tumor cells. Another lesion that was suspicious for FC by FNA was a nodular goiter with adenomatous hyperplasia confirmed by surgical pathology.

A limitation of our study is selective bias. This study is a retrospective study, and the subjects included were patients undergoing surgery. Whether the results are applicable to all clinical conditions remains to be verified. In addition, the strain ratio was not evaluated with ES. Further large studies using more indicators are needed to obtain a more accurate value.

## Conclusions

The CEUS enhancement pattern is helpful for differentiating between benign and malignant nodules with an intermediate or low suspicion. Heterogenous enhancement is associated with malignant nodules, a finding that could modify the clinical decision to avoid the misdiagnosis of FC in some patients. CUS characteristics, other qualitative CEUS indices, ES, and FNA have limited value.

## Data Availability Statement

All datasets generated for this study are included in the article/supplementary material.

## Ethics Statement

This prospective study was approved by the ethics committee of Peking Union Medical College Hospital, and informed consent was obtained. The patients/participants provided their written informed consent to participate in this study. Written informed consent was obtained from the individual(s) for the publication of any potentially identifiable images or data included in this article.

## Author Contributions

BZ and YJ conceived and designed the study. JX, RL, and RZ collected the data. XY and SZ performed the analysis. XL and XZ prepared all the figures and tables. XX, LG and QW were major contributors in writing the manuscript. SF edited the manuscript. All authors read and approved the final manuscript.

### Conflict of Interest

The authors declare that the research was conducted in the absence of any commercial or financial relationships that could be construed as a potential conflict of interest.
